# Dietary Polyphenols: Luteolin, Quercetin, and Apigenin as Potential Therapeutic Agents in the Treatment of Gliomas

**DOI:** 10.3390/nu17132202

**Published:** 2025-07-01

**Authors:** Weronika Justyńska, Mikołaj Grabarczyk, Ewa Smolińska, Aleksandra Szychowska, Andrzej Glabinski, Piotr Szpakowski

**Affiliations:** 1 Medical Faculty, Medical University of Lodz, 90-419 Lodz, Poland; weronika.justynska@stud.umed.lodz.pl (W.J.); mikolaj.grabarczyk@stud.umed.lodz.pl (M.G.); ewa.smolinska@stud.umed.lodz.pl (E.S.); aleksandra.bielenin@stud.umed.lodz.pl (A.S.); 2 Department of Neurology and Stroke, Medical University of Lodz, Zeromskiego 113 Street, 90-549 Lodz, Poland; andrzej.glabinski@umed.lodz.pl

**Keywords:** polyphenols, luteolin, quercetin, apigenin, gliomas, glioblastoma

## Abstract

Polyphenols are a group of plant-derived compounds that possess a wide range of possible industrial and pharmaceutical applications. Their mechanisms of action are often enabled by their multifaceted anti-inflammatory and antioxidant properties. As a result of their promising biological profile, they have been the focus of extensive research, which has examined their potential in the treatment of various diseases. These studies have observed that polyphenols may be associated with decreased neoplastic cellular growth, therefore offering valuable potential in oncological therapies. Quercetin, luteolin, and apigenin belong to the group of polyphenols with the most documented efficacy in this regard, particularly against tumors of glial origin. This review gathers information from a multitude of in vitro investigations and animal-model-based research that explore the molecular pathways and biochemical mechanisms engaged by polyphenols which enable their anti-tumoral activity in the central nervous system. Ultimately, this article aims to summarize this research and use this data to comment on the influence of polyphenols on glioma-affected subjects, in addition to exploring methods for increasing their bioavailability for the purposes of clinical application.

## 1. Introduction

The plant kingdom comprises a multitude of species, resulting in vast biodiversity with varied and complex chemical compositions. Some of these compounds have demonstrated an ability to influence the functionality of other types of organisms, such as animals, due to the structural analogies of their cytokines, hormones, and other messenger molecules. As a result, compounds derived from plants are often applied in pharmaceuticals or dietary supplements, as they demonstrate the potential to positively influence cellular metabolism and mitigate pathogenic threats. Of special interest for this review is one group of such phytochemicals—polyphenols [1,2]. Despite clear differences in the chemical structure of phytochemical compounds, all of them contain at least one aromatic ring and at least one hydroxyl group. More subtle characteristics determine their classification into subgroups of phenolic acids, stilbenes, and flavonoids. As of now, more than 8000 unique polyphenols have been identified, with more being documented continuously [3,4]. Although primarily recognized for their antioxidant and anti-inflammatory capabilities, extensive studies have noted their potential applications in the field of oncology, also [5]. Humanity is generally becoming older, and thus the prevalence and mortality rates of different oncological illnesses are rising. As a result, it is becoming increasingly important to search for new medications that could extend the lifespan of patients suffering from cancer. Cohort studies have analyzed the consumption of polyphenol-rich diets, observing that the application of the aforementioned polyphenols has the capacity to suppress cancer growth. These observations have been corroborated by data collected from neoplastic cell cultures, as well as tumor-affected animal models [6,7,8,9]. Such correlations have been noted in neoplasms of the liver, kidney, lung, bladder, bones, prostate, breast, colon, ovaries, and central nervous system (CNS) [10,11,12,13,14,15,16,17]. This review focuses exclusively on the studies concerning the CNS, specifically gliomas. Various polyphenols have been studied in the context of their application in the treatment of neurooncological disorders, and the best described include luteolin, quercetin, and apigenin, which are also the main focus of this paper. Luteolin (3′,4′,5,7-tetrahydroxyflavone) is a flavone present in carrots, peppers, celery, pomegranates, and herbs such as peppermint, rosemary, and parsley. It is recognized in the field of cancer research for its antioxidant activity, ability to suppress angiogenesis, and capacity to counteract tumor progression and metastasis. Furthermore, evidence suggests that it can enhance the sensitivity of neoplastic cells to apoptosis induced by cytotoxic drugs [18,19]. Quercetin (3,3′,4′,5,7-pentahydroxyflavone) is a flavonol that occurs naturally in various foodstuffs, including onions, apples, berries, and members of the cabbage family (*Brassicaceae*) [18,19,20]. Its potential anti-proliferative effects have been reported for various oncological disorders, including ovarian, lung, and colon cancer. In addition to this, it appears to exhibit synergistic effects with the classic chemotherapeutic drug cisplatin [21]. Apigenin (4′,5,7-trihydroxyflavone) is a flavone found in parsley, celery, peppermint, thyme, and chamomile [18,19,22]. In oncology, it has demonstrated the capacity to impede tumor progression by diminishing cancer cell motility and promoting apoptosis [23].

## 2. Characteristics of Gliomas and the Potential Place for Polyphenols in Their Treatment

The CNS comprises many different cell fractions, although the two most important ones are glial cells and neurons. The former constitute the most prevalent group and are, at the same time, the source of the most common type of CNS tumors—gliomas [24]. Because of their aggressive growth and unfortunate localization, which, in many cases, significantly limits the possibility of radical excision, they are among those with the worst prognosis for long-term survival. Depending on their histological features and resemblance to specific glial cell fractions, glial neoplasms can be classified as astrocytomas (the dominant type), oligodendrogliomas, or ependymomas [25]. It is also possible to classify glial-origin tumors depending on their localization in the brain or spinal cord, which mostly affects their symptomatic manifestation. Considering gliomas’ pathological growth and dysplastic characteristics, the World Health Organization (WHO) distinguishes four grades of their histological malignancy, with grade IV being the most serious and associated with the highest mortality and worst survival rate, as most patients do not live longer than 2 years after being diagnosed. Grade IV gliomas are described as glioblastomas (GBMs). Unfortunately, GBM is the predominant type of newly diagnosed malignant CNS neoplasm. Two age groups are at the highest risk of developing such a tumor, the first one being children and the other one being elderly patients, particularly those in their eighth decade of life [26,27]. As mentioned before, due to their localization in the CNS, the radical excision of gliomas is not always possible, as it may be associated with an increased risk of damaging structures crucial for survival, which are responsible for maintaining basic life processes like breathing, thermoregulation, and the control of blood pressure and heartbeat. Despite this, neurosurgical procedures and radiotherapy are still the main therapy form and offer patients the best chance for survival [28]. However, this draws attention to the need to find alternatives in the form of new medicines that could directly eliminate glioma cells or work as supportive drugs to maximize the effect of other treatment protocols with already proven effectiveness, like the usage of temozolomide (TMZ) [29]. This could increase the chances for patients who could not be qualified for surgical approach or with tumor remnants still present after excision. Polyphenols, especially quercetin, luteolin, and apigenin, form a promising group of compounds in this matter due to their wide plain of anti-tumoral capabilities and minimal toxicity towards normal cells. Numerous in vivo studies show that the abovementioned phytochemicals are able to induce apoptosis in different gliomas and counteract angiogenesis, leading to the diminution of tumor volume [30,31,32]. Some of the mechanisms leading to such outcomes include the activation of the MAPK pathway, induction of mitochondrial oxidative stress, stimulation of different caspase proteins, modulation of the chaperones, and promotion of microglial response towards neoplastic cells [33,34,35,36].

## 3. Natural Sources, Absorption, Metabolism, and Novel Pharmaceutical Measures to Increase Polyphenol Bioavailability

### 3.1. Luteolin

Luteolin is classified as flavone, a subtype of the flavonoid family. Similarly to quercetin, it is composed of two benzene rings linked by an oxygen-containing pyrene ring. Its biological activity is mostly attributed to the presence of four hydroxyl groups and a double bond existing between carbon atoms C2 and C3 of the whole particle [37]. Naturally, it is usually found in a glycoside form, with luteolin-7-O-glycoside being the most frequent one. Most common dietary sources of this polyphenol include peppermint, broccoli, carrots, thyme, oregano, or Chinese celery. The bioavailability of luteolin, upon oral intake, is relatively low and oscillates in different studies between 4 and 25% [38]. Interestingly, the previously mentioned luteolin-7-O-glycoside demonstrates lower oral bioavailability than the unmodified form, and most of its contents are primarily hydrolyzed to luteolin in the gastrointestinal tract, and only then are they absorbed [37]. In order to circumvent the problem of unsatisfactory bioavailability, parenteral supply methods, like intravenous or intraperitoneal injections, can be used. Such solutions are frequently utilized in studies that assess luteolin’s capabilities in the treatment of various diseases in animal models. However, this route of administration still does not eliminate the issue of the relatively rapid metabolism of phytochemicals, which mostly occurs in the liver. Hepatocytes can subject luteolin and its derivates to methylation, sulphation, or glucuronidation processes, and the obtained metabolites may enter systemic circulation, undergo enterohepatic cycling, or be eliminated [38]. In order to maximize luteolin’s potential as plant-derived medication, it is crucial to ensure its constant concentration in body fluids and, most importantly, its targeted supply to the tissues affected by disease processes, which, in terms of this review, means malignancies of glial origin located in the CNS. Similarly to quercetin, here, too, the leading strategies are those based on universally used carrier compounds, such as liposomes or nanoconjugates [37]. Methoxypolyethyleneglycol-b-polycaprolactone (MPEG/MCL) micelles allow for the improved cellular uptake of luteolin and, because of this, increase its toxicity towards malignant cells [39,40]. Bioavailability can also be raised by modifying luteolin crystals’ surface with sodium dodecyl sulfate (SDS), which serves as an opening factor for tight junctions—structures that enhance biological barrier integrity [41]. Another approach involves bilosomes—conjugates of bile and phospholipids—as they upregulate the fluidity of biological membranes, including the BBB. Trials performed on mice prove that the intranasal administration of luteolin in bilosomal form greatly adjusts the efficacy of therapies that focus on delivering this phytochemical directly to the CNS [42]. To achieve a more stable release of luteolin—and, through this, ensure its constant presence in plasma at biologically active concentrations—the liposomes may be coated in chitosan and be applied intranasally. This improves their stability and, as chitosan forms gel-like structures in the nasal cavity that are quite resistant to mucociliary clearance, the absorption from nasal mucosa can be sustained at a satisfactory level for a prolonged time [43].

### 3.2. Quercetin

Quercetin belongs to the flavonol subclass of flavonoids. Chemically, it is composed of two benzene rings and a pyrene ring that contains oxygen. Its biological activity is attributable mostly to the presence of five hydroxyl groups. Typically, it occurs in glycoside form, where at least one of those groups is occupied by a sugar substituent. This polyphenol can be isolated from a wide variety of different dietary products of plant origin, including onions, cabbages, tomatoes, cocoa, lettuce, radish, pepper, blackcurrant, figs, and many other fruits or vegetables [44,45]. Despite the promising results from studies proving its therapeutic capability in different illnesses—most importantly, oncological ones—there are still many obstacles to overcome in terms of actually introducing this substance as potential medication. Most problems are connected with weak solubility in water, the poor bioavailability of unmodified molecules, instability to oxidants, and biotransformation in metabolic processes [46]. If administered orally, quercetin can be subject to modifications, as, in the acidic environment of the stomach, some part of the applied dose is degraded to phenolic acids. In the intestines, the efficacy of its absorption depends mostly on the specific glycoside form. Quercetin-3-O-oligoglucosides exhibit 10 times higher bioavailability than quercetin-3-O-rutinosides and 20 times higher bioavailability than quercetin aglycones [45,47]. The absorbed molecules can pass further alterations in the small intestine, colon, liver, and kidneys, leading to quercetin glucuronidation, methylation, and sulfation. The analyses show that, after consuming food rations composed of products with high quercetin content, the total plasma concentration of this phytochemical and its derivates does not exceed 200 nmol/L, which is not enough to exert a strong inhibitive effect on the growth of various malignant cells [45]. Animal models prove that higher levels can be achieved by repetitive administration of adequately condensed quercetin solutions or extracts from plants with exceptionally rich deposits of this phytochemical, although this can lead to the increased occurrence of adverse effects, mostly from the gastrointestinal system [44,45,48]. Considering this, most strategies aimed at raising quercetin’s efficacy in the potential treatment of various disorders, not only oncological ones, focus on altering quercetin particles’ structure or on increasing their bioavailability by packing the molecules in different carriers or fusing them with other substances that serve as grip points for specific target tissues. Parenteral administration of this phytochemical eliminates the problem of low oral bioavailability; however, the issue of quick metabolic modulation and elimination remains [49]. Systems utilizing carrier molecules mostly include liposomes, polymeric micelles, polylacticoglycolic acid (PLGA) nanoparticles, particles based on biomacromolecules like albumines or lactoferrin, and carbon- or metal-oxide-containing conjugates [45,46]. Strategies of this sort greatly increase quercetin’s stability in different conditions of pH, temperature, or UV exposition. Moreover, they allow for the achievement of a more stable pharmacokinetic profile in body fluids, as well as the significant upregulation of cellular uptake, which are all demanded alterations for possible clinical translation [46]. When considering quercetin as potential drug for treatment of CNS tumors, especially gliomas, an important factor is also the ability to cross the BBB. This is a crucial process for achieving a biologically effective concentration in brain and spinal cord tissues and counteracting the growth of glial malignancies. Similarly to their absorption at the intestinal level, some analogs of quercetin are structurally predisposed and pass through the BBB more efficiently. However, regardless of this, their application without additional modifications or utilization of specific carriers still leads to relatively low absorption and greatly limits the full potential of this polyphenol in the treatment of CNS disorders [50]. The intranasal route of administration seems promising as a convenient alternative to oral supply, as the high permeability of the nasal mucosa, along with its extensive vascularization, enable the efficient systemic absorption of applied polyphenol, especially when quercetin is conjugated with solubility-enhancing factors like β-cyclodextrin derivatives [51]. As biological barriers have numerous similarities, the previously mentioned transport systems, like liposomes, various nanoparticles, or biomacromolecules, upregulate both intestinal absorption and penetration of the BBB by quercetin molecules that are connected with them [52]. This can be furtherly optimized by adding molecular anchors specific for channel structures present in the BBB. Attaching glucose to quercetin-containing liposomes allows them to take advantage of glucose transporter-1 (GLUT-1), a membrane protein responsible for obtaining blood glucose for brain tissues [53]. Nanoconjugates can also benefit from including rabies virus glycoprotein (RVG29) in their structure, as it shows an affinity to the nicotinic acetylcholine receptor present in the BBB [54]. Another interesting approach involves the use of superparamagnetic iron oxide nanoparticles (SPIONs). In the presence of an external magnetic field, they release the attached polyphenol in a controlled manner, which can be used to deliver quercetin directly to CNS [52]. Similar mechanism is utilized in zeolitic imidazolate framework 8-coated Prussian blue nanocomposite (ZIF-8@PB); however, in this case, the release of quercetin is dependent on its exposure to near-infrared radiation (NIR) [55].

### 3.3. Apigenin

Apigenin is a flavone compound widely distributed in the plant kingdom. Among food products with a high apigenin content, the following deserve special attention: parsley, celery, onions, oranges, wheat sprouts, peppermint, thyme, and oregano. As for its chemical structure, it shares similar carbonic skeleton with other flavonoids, being composed of two benzene rings connected by a three-carbon chain that forms a heterocyclic oxygen-containing ring. The number of hydroxyl groups attached to the whole structure is three. In plant tissues, it predominantly exists as O- or *C*-glycosides. The most common ones include apigenin-7-O-apiosylglucoside, apigenin-7-O-glucoside, apigenin-8-*C*-glucoside, and apigenin-6-*C*-glucoside [56]. In the gastrointestinal tract, about 5–10% of apigenin present in consumed food can be absorbed, reaching the blood and, with it, the main metabolic hub of the body, which is the liver. Analyses performed on rodent models are inconsistent in terms of absorption’s speed, and demonstrate that it may significantly vary between different animal species and depend on microbiota profiles. The main products of the hepatic and intestinal metabolism of apigenin are its sulfated and glucuronic acid derivates, as well as luteolin. After consuming plants with high apigenin content, the mean concentration of apigenin and its analogs in the blood plasma reaches values of approximately 127 nmol/L. Their further transport to specific tissues is determined mostly by their expression of lipoprotein receptors and cholesterol carriers, as these affect the cumulation of luteolin [57]. As is typical for polyphenolic compounds, the literature agrees that the overall bioavailability of orally ingested apigenin in its native form is poor, and this is the major factor limiting its use as an effective potential drug. However, the direct admission of this phytochemical in a parenteral way allows it to bypass the problem of poor absorption and at least partially counteract its metabolic alterations as the enterocyte processing step is skipped [58]. Similarly to luteolin and quercetin, various forms of transport systems are used to improve the effectiveness of apigenin as a protective and therapeutic factor. Among the relatively simple solutions are the conjugates of this phytochemical with different amino acids, which significantly improve its capability to cross biological barriers, including the BBB [59]. Other possible conjugates with more favorable pharmacokinetic profiles are apigenin monophosphates [60]. More complex methods involve the use of liposomes, hydrogels, nanostructured lipid carriers, microemulsions, nanoemulsions, and emulsions, that apart from improving bioavailability and providing stable distribution, also increase apigenin’s stability in conditions of different temperatures or exposition to oxidants [57].

## 4. Luteolin’s Role in the Treatment of Gliomas

### 4.1. Reports from In Vitro Studies on Luteolin Activity to Glioma Cells

**Figure 1:**
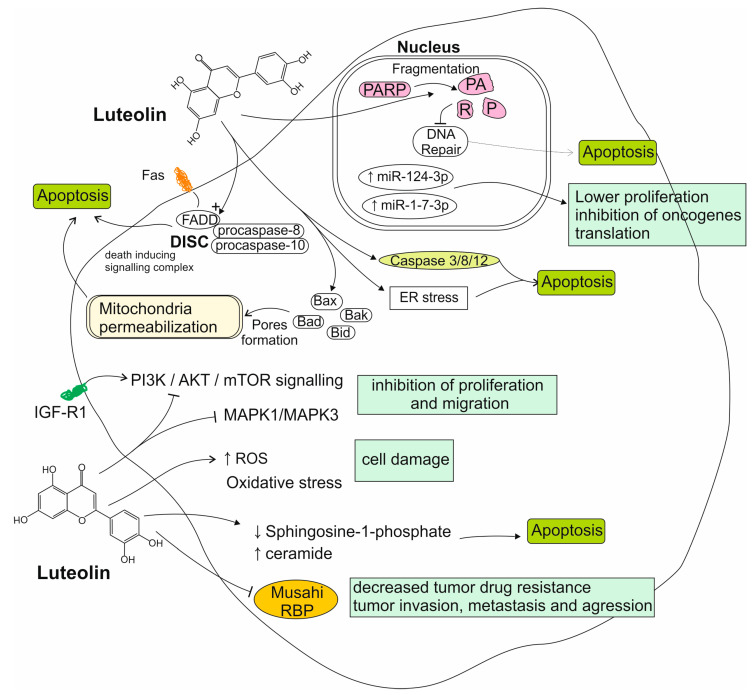
Summarized mechanisms of luteolin activity toward glioma cells. ↓—decreased activity/amount, ↑—increased activity/amount.

Luteolin, as well as its two most common glycosides—luteolin-7-*O*-glucoside and luteolin-8-*C*-glucoside—have been found to induce cell cycle arrest and apoptosis in GBM cells in a caspase-3-dependent manner, with elevated levels of caspase-3, caspase-8 and poly ADP ribose polymerase (PARP), as well as the pro-apoptotic proteins Bak, Bid, Bax, and Bad being observed [61,62,63]. It has been reported that luteolin decreased proliferation by regulating the Akt and MAPK signaling pathways. These are overly active in GBM due to the overexpression of the epidermal growth factor receptor (EGFR) [64]. Analyses performed on three different glioma cell lines, U251, LN229, and SNB19, prove that an even stronger and more efficient suppression of the mentioned Akt pathway can be achieved by combining luteolin with valproic acid—a classic anticonvulsant drug [65]. Powe et al. have additionally found that luteolin exhibits a synergistic anti-proliferative effect with the EGFR inhibitor-erlotinib, which has been studied in clinical trials for the treatment of GBM [66]. The anti-proliferative properties of luteolin can be attributed to the induction of oxidative stress and mitochondrial dysfunction, also [67]. Different studies describe that luteolin is capable of reducing GBM cell migration and the formation of new clusters of malignant cells. This inhibitive effect occurs via the insulin-like growth factor 1 (IGF-1) receptor and the downstream PI3K/Akt/mTOR signaling pathway, consequently inhibiting the process of epithelial–mesenchymal transition (EMT), which is responsible for the increased motility and invasiveness of GBM cells [33,68,69]. Another molecular way for luteolin to promote dysplastic cells death is via the extrinsic apoptosis pathway, which includes the FAS-associated death domain protein (FADD) cell death receptor. This mechanism comes with elevated levels of caspase-3, caspase-8, and PARP. Luteolin also upregulates the expression of the tumor suppressor miRNAs, miR 124-3p, and miR-1-7-3p. This suggests a link between these specific miRNAs and luteolin’s anti-proliferative properties against glioma [33,70,71]. Additionally, Chakrabarti et al. have found that luteolin and silibinin (another plant-derived flavonoid) exhibit a synergistic anti-glioma effect which is more potent than the conventional chemotherapeutics (temozolomide and carmustine) used in monotherapy [72]. Using molecular docking technology, Huang et al. identified six core targets of luteolin activity against glioma, including AKT, MAPK3, MAPK1, and tumor necrosis factor (TNF) (in agreement with the aforementioned studies). Additionally, the transcription factor JUN has emerged as a previously unsuspected possible luteolin target [73]. The described phytochemical regulates the activity of the RNA-binding protein-Musashi1. This has been implicated in the tumorigenesis of various malignancies, including GBM. This protein has also been found to correlate with the resistance of tumor cells in chemo- and radiotherapy [74]. Luteolin has also been found to induce autophagy in GBM cells, which, in some conditions, can promote either cell survival or increase the chances of cell death. The induction of autophagy interferes with the induction of apoptosis by luteolin, diminishing the compound’s anti-proliferative effect. The autophagy inhibitor 3-methyadenine (3MA) was found to effectively increase luteolin-induced apoptosis in GBM, promising a potential combined strategy [75]. In a recent study by Navone et al., it has been observed that luteolin affects the production of the sphingolipid metabolite sphingosine-1-phosphate (S1P), a molecule which has been studied as a cancer biomarker and is reported to play a role in promoting tumor genesis and invasion. Luteolin is able to shift the balance between S1P and ceramide towards increased ceramide levels, resulting in an anti-tumoral effect [76]. Figure 1 summarizes mechanisms of luteolin activity toward glioma cells.

### 4.2. Reports from Animal Models on Luteolin Activity

In agreement with in vitro findings, luteolin has been found to induce the apoptosis of GBM cells in xenograft-bearing mice, reducing tumor volume without causing bodyweight loss or any apparent hepatotoxicity. The apoptotic effect can be attributed to reactive oxygen species (ROS) production, mitochondrial dysfunction, and endothelial reticulum (ER) stress, along with the induction of cleaved-caspase-12 and caspase-3 being observed [67]. In attempts to increase luteolins’ poor bioavailability, Zheng et al. have developed nanomicelles containing this polyphenol which allow for improvements in the compound’s solubility in water and which prolong its presence in the plasma and enhance anti-tumoral efficacy in GBM-bearing mice [30]. Another approach involves nanoformulations containing luteolin, previously modified with folic acid, a natural ligand of folate receptors, which have a low expression in normal cells, but which are frequently overexpressed in tumor cells. Such combination promotes luteolin’s antiproliferative effect against glioma in mice, both in subcutaneous and intracranial glioma models [32]. However, further investigation is needed before the challenge of poor bioavailability can be effectively overcome and luteolin can find a clinical application in the treatment of CNS tumors. Results from studies investigating the effect of luteolin on gliomas in vivo studies are summarized in Table 1.

## 5. Quercetin’s Role in the Treatment of Gliomas

### 5.1. Reports from In Vitro Studies on Quercetin Activity to Glioma Cells

**Figure 2:**
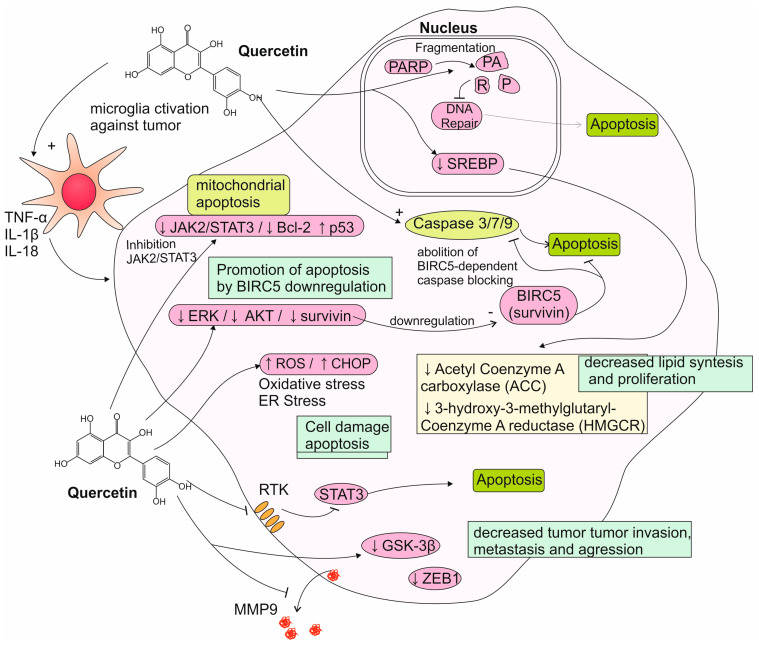
Summarized mechanisms of quercetin activity toward glioma cells. ↓—decreased activity/amount, ↑—increased activity/amount.

Multiple studies have attempted to elucidate the mechanisms responsible for the effects of quercetin. Said effects are reported to be as follows: apoptosis, a decrease in proliferation, and induction of cell cycle arrest in human glioma cell lines [77]. It has been suggested that quercetin induced apoptosis in ROS-dependent and ROS-independent pathways by downregulating the ERK, AKT, and anti-apoptotic protein survivin, therefore resulting in caspase-dependent cell death [78,79]. Elevated activity levels of caspase-3, -7, and -9 have been observed in quercetin-treated glioblastoma cells, accompanied by an overexpression of cleaved PARP. The apoptotic effects of quercetin on some glioblastoma cells have also been linked to ER stress and oxidative stress, showing elevated ROS levels and an increased expression of ER-stress-related molecules, such as the CHOP transcription factor and the *Atf4* and *Atf6α* genes [80,81]. It has also been observed that, at lower concentrations, quercetin inhibits the JAK2/STAT3 pathway, while higher concentrations of the drug cause a downregulation of the anti-apoptotic Bcl-2 family proteins, increase p53 expression, and lead to apoptosis in the ROS-related mitochondrial pathway [82]. Another study noted that the induction of apoptosis via the STAT3 pathway inhibition is tied to the Axl receptor, which is overexpressed in GBM cells [83]. Additionally, quercetin has been found to inhibit the expression of metalloproteinase (MMP)-9 and fibronectin, two extracellular matrix proteins that are associated with glioma progression [84]. Quercetin has also been observed to affect the anti-apoptotic chaperone protein known as heat shock protein-72 (Hsp72), which is often overexpressed in tumor cells and has been linked to tumor chemoresistance. Quercetin causes Hsp72 to migrate into the nucleus and exert a protective effect on nucleal structures, which could contribute to increased cell survival and chemoresistance in anaplastic astrocytoma cells [85,86,87]. Silencing Hsp72 with specific siRNAs improves the efficacy of quercetin in combination with both temozolomide and sorafenib against astrocytoma and GBM [35,85]. The selective esterification and bromination of quercetin produce derivatives, which exert a stronger cytotoxic effect and a higher degree of selectiveness towards glioma cells than sole quercetin does [88]. Quercetin has also been found to downregulate the expression of the transcription factors of the sterol regulatory element-binding protein (SREBP) family, which promote the de novo synthesis of fatty acids, triglycerides, and cholesterol. Quercetin-treated glioma cells display lower activity of enzymes involved in the synthesis of these lipids (including HMG-CoA reductase (HMGCR) and acetyl-CoA carboxylase (ACC)). Since a highly effective synthesis of lipids is needed for the mebranogenesis of proliferating cells, an inhibition of this process may be partially responsible for quercetin’s anti-proliferative action against gliomas. However, the exact significance of this process in the polyphenol’s overall anti-tumoral potential remains unclear [89]. Additionally, treatment with quercetin and its related flavonoid rutin modulated the inflammatory response of microglial cells, shifting their profile towards the expression of IL-1B, IL-18, and TNF, while decreasing the levels of the oncogenic IL-6. In microglia/glioma co-cultures, the flavonoids inhibited the proliferation and migration of the neoplastic cells [90]. In recent studies, it has been found that quercetin inhibits the GSK-3β/β-catenin pathway, consequently downregulating the ZEB1 transcription factor, which is involved in epithelial–mesenchymal transformation—a process crucial for increasing the motility, invasiveness, and metastasizing potential of glioma cells [91]. Quercetin has also been studied in conjunction with other substances that may act synergistically against glioblastoma. The combination of quercetin and sodium butyrate (a histone deacetylase inhibitor) has been found to inhibit protective autophagy, a degradation process which allows for tumor cells to survive in spite of nutrient deprivation or cancer treatments. Quercetin and sodium butyrate exhibited synergistic action, inducing apoptosis in both rat and human glioblastoma cells. This effect may be beneficial in overcoming resistance to chemotherapeutics such as temozolomide [92]. Another interesting development may be the synthesis of a quercetin-losartan hybrid molecule, which utilizes losartan’s inhibition of the AT1R angiotensin receptor and quercetin’s antioxidative properties to reduce tumor proliferation and inhibit angiogenesis [93]. Mechanisms of quercetin action toward glioma cells were summarized on Figure 2. Despite these promising findings, quercetin’s poor bioavailability and difficulty crossing the BBB have proved a significant challenge in the transition to the clinic. In search of clinically applicable drug delivery systems, several types of nanoformulations have been developed. These include freeze-dried nanomicelles and magnetoliposomes loaded with quercetin, both of which are successful in reducing the viability of C6 glioma cells in vitro [91,94]. Ersoz et al. have developed four nanoparticles of varied size using a polymer of a lactic acid derivate loaded with quercetin. They have found that the smallest particle containing 25 mg of quercetin and measuring 215.2 nm was the most effective at permeating glioma cells and exerting an anti-tumoral effect [95]. In a recent study, Soriano-Ursua et al. decided to examine a different approach and developed nanoliposomes loaded with quercetin in conjunction with 3-bromopyruvate (3-BP), a molecule which exhibits strong alkylating properties and cytotoxic activity towards various cancer cells through ATP depletion. This formulation has been successful in inhibiting astrocytoma growth in vitro as well [31].

### 5.2. Reports from Animal Models on Quercetin Activity

The results of in vivo studies on the effect of quercetin on gliomas suggest that some caution should be taken before clinical application. Contrary to in vitro findings, one in vivo study reported that quercetin increased the tumor size of C6 gliomas in a rat model. The mechanisms responsible for this effect are unclear, but the authors pointed to a reduced infiltration of lymphocytes into the tumor and slightly reduced systemic T-cell proliferation, suggesting an impaired immune response to the tumor [96]. Brain tissue analysis following systemic administration revealed quercetin concentrations of 0.16 µg/g (~530 nM)—substantially lower than those used in vitro [97]. When lower physiologically relevant doses were tested in vitro, concentrations below 10 µM failed to significantly inhibit glioma colony formation, highlighting a disconnect between in vitro efficacy and achievable in vivo levels. The lack of tumor cell depletion at low concentrations of quercetin may lie in its multifaceted biological activity. As it is known, apart from being an antitumoral agent, quercetin displays numerous beneficial effects towards different cell’s metabolism and functionality. It is possible that, in such low concentrations, the cytostatic effect becomes overshadowed by those positive alterations, and the net effect is an increased proliferation instead of cell growth suppression [82]. Contrary to those findings, Chen et al. have reported that quercetin suppresses the GSK-3β/β-catenin/ZEB1 pathway in murine glioma xenograft models, resulting in a downregulation of EMT markers. The reduction in the EMT process has been associated with a decrease in tumor volume in mice. Additionally, mice treated with quercetin displayed lower weight loss than mice treated with TMZ—a standard chemotherapeutic in terms of glioma treatment—suggesting a low toxicity profile [98]. Attempts to enhance quercetin’s poor physicochemical properties and develop effective drug-delivery systems include freeze-dried quercetin-loaded nanomicelles (FD-NMs) and nanoliposomes (NLs) containing quercetin and 3-BP. Both approaches are successful in accumulating the phytochemical in the tumor tissue of glioma-bearing mice, improving the animals’ survival time and decreasing tumor volume [31,91]. Nanoparticles composed of quercetin and stabilized with amphiphilic polymers reach CNS structures affected by malignancy 18 times more effectively than in cases of tumor-free brains. Moreover, in a mice GL261 syngeneic glioma model, as well as human PS30 glioblastoma xenografts, they exhibit a significant antiangiogenic impact, greatly disrupting the formation of tumor vessels through the inhibition of vascular endothelial growth factor receptor 2 (VEGFR2) function [99]. Results from studies investigating the effects of quercetin on gliomas in vivo studies are summarized in Table 2.

## 6. Apigenin’s Role in the Treatment of Gliomas

### 6.1. Reports from In Vitro Studies on Apigenin Activity to Glioma Cells

**Figure 3:**
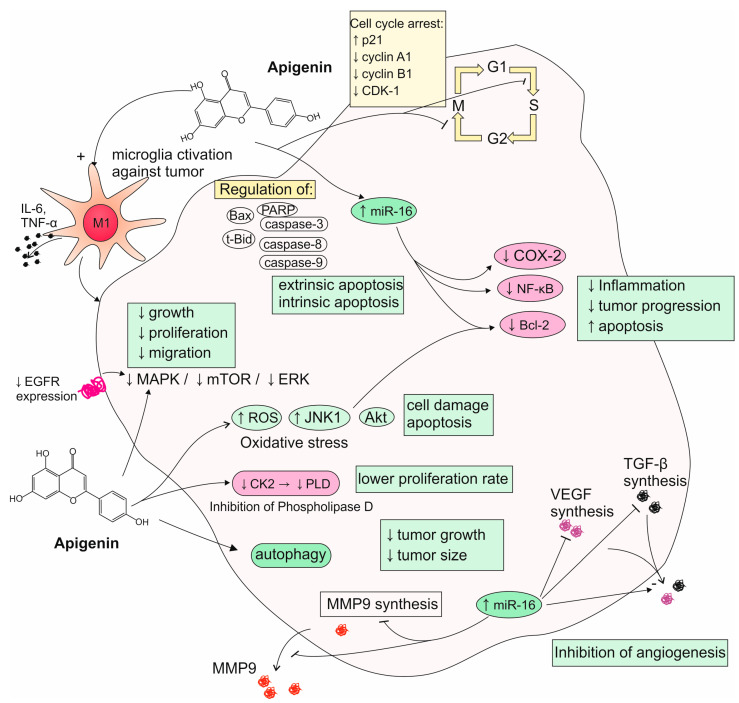
Summarized mechanisms of apigenin activity toward glioma cells. ↓ means decreased; ↑ means increased.

Apigenin has been observed to exhibit anti-tumoral properties in various neoplasms in vitro. Recently, it has been found that apigenin exerts an anti-migratory effect on C6 glioma cells and can reduce their viability and proliferation rate in a time-dependent and dose-dependent manner. The exact mechanisms responsible for such effects may be explained by the alteration of IL-6 cytokine levels and the potential of activating microglia chemotaxis towards the glioma, restoring the antitumor immune response. Simultaneously, apigenin promotes a proinflammatory M1 microglia profile, most likely by altering the production of IL-10 and TNF [36]. Treatment with this flavone also significantly increases the production of nitric oxide, known for its inflammatory capabilities, improving the immune response against the tumor [100]. In contrast, Mazzio et al. have identified apigenin as exhibiting potent anti-inflammatory activity in lipopolysaccharide (LPS) and interferon-γ (IFN-γ)-activated C6 glioma cells, as it proves capable of downregulating iNOS expression and counteracting cytokine-induced neutrophil chemoattractant-3 (CINC-3) release [101]. The observed discrepancy may come from the distinct basal conditions in the cellular environment or differences in the applied concentrations of tested phytochemical. As other studies show, the concentration range at which apigenin causes the loss of glioma cells’ viability is quite wide and covers the values between 1 and over 30 µg/mL. Unfortunately, this tumor-suppressing effect is reversible if the cells are reintroduced into apigenin-free culture medium [100,102,103,104]. What is important about this compound is that it has been reported to cause apoptosis in GBM cells without affecting the normal astrocytes, indicating selective action for controlling such neoplasms. A possible mechanism is apoptosis induced by the ROS-dependent pathway in connection with the activation or phosphorylation of the JNK1 protein. Furthermore, apigenin downregulates the expression of anti-apoptotic protein Bcl-2 and counteracts the activation of the key anti-apoptotic kinase Akt, which are overly active in GBM. Moreover, it inhibits the EGFR-mediated activation of MAPK protein kinase and mammalian target of rapamycin (mTOR), as well as opposes phosphorylation of ERK. Those molecular targets are responsible for the enhancement of cell growth, proliferation, differentiation, and migration; thus, their suppression is associated with a potential anti-tumor effect [105,106]. Ahn et al. report yet another mechanism, as apigenin diminishes phospholipase D (PLD) activity, critical for U87 glioma cells’ multiplication, by targeting casein kinase II (CK2) [107]. Apigenin has been found to arrest the cell cycle in the G0/G1 phase and G2/M phase by degrading or inhibiting the expression of corresponding cyclins and cyclin-dependent kinases (CDKs) [100]. G2/M phase cell cycle arrest in GBM cells is achieved through mechanisms, including ROS-inducted oxidative stress, as well as the upregulation of p21 and downregulation of cyclin-A1, cyclin-B1, and CDK-1. It has been found that the discussed flavone promotes both extrinsic and intrinsic apoptosis pathways by regulating Bax, t-Bid, caspase-8, caspase-9, caspase-3, and PARP (Shendge et al., 2021) [34]. Apart from favoring apoptosis, apigenin can stimulate autophagy, also, by upregulating its markers like LC3-II and beclin-1 while reducing p62 levels [108]. Wätjen et al. highlight that even stronger outcomes regarding apoptosis induction can be achieved with C8-prenylated apigenin derivatives, mostly via its effects exerted on caspase-3 and caspase-7 [109]. Anti-tumoral properties of apigenin may also be attributed to the immunomodulatory ability of suppressing the expression of dysregulated cyclooxygenase-2 (COX-2) and NF-κβ, which are both associated with inflammation and cancer progression [105]. In the process of neoplasm formation, an important element is angiogenesis, which allows for the tumor to be provided with a suitable influx of nutrients crucial for its further growth. Studies performed on U343 and U118 glioma, as well as the GL-15 glioblastoma cell line, show that apigenin negatively modulates the secretion of two cytokines important for the origination of new vessels—vascular endothelial growth factor (VEGF) and transforming growth factor-β1 (TGF-β1) [110,111]. The most important mechanisms of apigenin activity toward glioma cells are summarized in Figure 3. Currently, more and more attention is being paid to the role of miRs in modulating basic metabolic processes and their importance in various diseases, including oncological ones. Apigenin has the capability to alter the expression of the above-mentioned molecules, which is also most likely important for its overall anti-tumoral potential. Recent research has demonstrated that the discussed phytochemical significantly upregulates the expression of tumor-suppressive miRs, such as miR-16, which stifle oncogenic targets like Bcl-2, NF-κβ, and MMP-9, leading to the reduced invasion and increased apoptosis of glioma cells [106,112]. Santos et al. have demonstrated that apigenin modulates MMPs activity by reducing their overall expression while simultaneously sparing active MMP-2 levels. This is accompanied by a diminution of human GL-15 glioblastoma cells’ migrative and invasive potential. In addition to preventing the degradation of the extracellular matrix, apigenin may also contribute to its synthesis, among others, by increasing the production of fibronectin [113]. GBM stem cells have been recognized as a primary cause of GBM development, therapy resistance, and tumor recurrence. Targeting *c*-Met signaling, crucial for maintaining the GBM stem-like phenotype, might be a promising strategy to improve the outcome of GBM patients by eliminating GBM stem cells. It was found that apigenin administration significantly reduces GBM stem-like cells’ capacity for self-renewal and invasiveness, most likely by affecting the *c*-Met-mediated signal transduction pathways. Also, their molecular markers, such as CD133, Nanog, and Sox2, show decreased expression levels due to apigenin’s suppression of *c*-Met signaling [114,115]. Another plain for apigenin to be considered as a potential medical agent is radiotherapy treatment, as its possesses an ability to sensitize gliomas to radiation. The mechanism of radiosensitization after apigenin addition to a culture medium is possibly associated with the reduction in hypoxia-inducible factor-1α (HIF-1α) expression, which might subsequently downregulate the downstream glucose transporter (GLUT)-1/3, NF-κβ, and pyruvate kinase isozyme 2 (PKM2). This results in the attenuation of glycolysis and prevents lactic acid (LA) from being synthesized. As high levels of LA are associated with greater radio resistance, such metabolic alteration can be responsible for an apigenin-dependent rise in the tumor’s susceptibility to radiation [116]. In contrast, Kroonen et al. indicate that, while apigenin negatively modulates casein kinase (CK2) pathways, a key regulator of cell proliferation and the DNA damage response, it does not enhance glioma cell radiosensitivity. In their study, despite causing the significant inhibition of CK2, apigenin was unsuccessful in impairing double-strand DNA break repair or sensitizing glioma cells to γ-ray cytotoxicity [117]. Apart from radiotherapy, a second acclaimed approach for the treatment of gliomas is chemotherapy, with TMZ considered to be the current golden standard. Wang et al. have found that treatment composed of combined TMZ and apigenin may lead to better outcomes, at least in in vitro trials. Compared to standalone drugs, the combination of the two more efficiently decreases the proliferation of glioma cells and triggers their arrest at the G2 phase. It also markedly inhibits the protein expression of p-AKT, cyclin D1, Bcl-2, MMP-2, and MMP-9 [118]. Apigenin can also work synergistically with the other phytochemicals broadly examined for their possible utilization in glioma treatment strategies. There are reports of its synergistic or sensitizing potential when applied simultaneously with hydroxygenkwanin, luteolin, quercetin, baicalin, chrysin, and scutellarein for fighting neoplastic cells of various glioma lines [102,119,120]. However, some combinations may not be beneficial, as Teles et al. report that co-treatment with acacetin and apigenin lacks toxicity towards UVW glioma cells [121]. These results highlight the complexity of flavonoid interactions in oncological treatment and suggest that mixing flavonoids may require precise optimization to achieve enhanced therapeutic efficacy.

### 6.2. Reports from Animal Models on Apigenin Activity

As for its potential effectiveness in CNS tumors, in vivo findings have been inconclusive. Contrary to in vitro results, a study by Engelmann et al. showed that apigenin injections were ineffective in diminishing the size or vascularity of established tumors. To be more specific, apigenin had a slight effect on the mean size of the slowly growing C6 gliomas in mice. The authors suggest that apigenin might have reduced angiogenesis, but not enough to retard the formation of tumors, and it is potentially explainable by the stochiometric insufficiency of the employed dose, the weakening of the non-specific immune defense secondary to the anti-inflammatory properties of apigenin, or the upregulation of matrix-degrading enzymes other than hyaluronidase [104]. A different study that examined the apigenin as a radiotherapy-sensitizing agent in mice showed the efficiency of a 20 mg/kg dose in increasing the loss of glioma volume among animals submitted to 8 Gray irradiation. The authors attribute such a phenomenon to apigenin’s ability of suppressing glycolysis in glioma tissues by inhibiting the activity of hexokinase (HK), phosphofructokinase (PFK), pyruvate kinase (PK), and lactate dehydrogenase (LDH) and the expression levels of GLUT-1/3 and PKM2. This leads to a lower accumulation of LA, which serves as a factor that is attributed to causing radio resistance [122]. Results from studies investigating the effect of apigenin on gliomas in in vivo studies are summarized in Table 3.

## 7. Conclusions and Future Perspectives

Quercetin, luteolin, and apigenin have demonstrated notable antitumoral potential against glioma in both in vitro and in vivo studies. However, their clinical application remains limited due to several key pharmacokinetic challenges, including poor aqueous solubility, low bioavailability, limited membrane permeability, and chemical instability—all of which hinder their effective transport across the BBB. These limitations are particularly critical in therapeutic contexts that require sustained drug concentrations at the tumor site, such as senescence-inducing treatments.

Pharmacokinetic and pharmacodynamic modeling may help to define optimal dosing strategies and facilitate the achievement of clinically relevant systemic concentrations. To address these limitations, various advanced drug delivery systems have been investigated, including freeze-dried nanomicelles, magnetoliposomes, and PLGA-based nanoparticles. These approaches aim to improve solubility, bioavailability, and the controlled release of quercetin, luteolin, and apigenin. Further studies of such delivery strategies may enhance the therapeutic efficacy of polyphenols and other hydrophobic compounds, broadening their potential clinical applications. In parallel, a deeper understanding of structure–activity relationships is required to identify the molecular features that influence the potency, selectivity, and BBB permeability of polyphenol derivatives and their carrier systems. Another promising approach is the utilization of synergistic treatment combinations. Given gliomas’ resistance to conventional therapies and their high recurrence rates, it is essential to investigate whether these polyphenols can enhance the sensitivity of tumor cells to radiotherapy and chemotherapy over a prolonged treatment course. Moreover, luteolin should be evaluated not only for its direct cytotoxic effects, but also as a sensitizing agent that could enhance the efficacy of standard therapies or reduce their required dosages. Despite emerging data, no studies have, as yet, comprehensively assessed long-term outcomes such as tumor recurrence, the development of treatment resistance, or the effects on dormant tumor cell populations. Future research should also explore the modulation of microRNAs by polyphenols. The ability of luteolin and apigenin to influence miRNA expression offers a novel mechanistic dimension. As key regulators of post-transcriptional gene expression, microRNAs may serve as critical intermediaries between dietary compounds and tumor signaling networks. The systematic profiling of miRNA responses to polyphenol exposure could reveal novel therapeutic targets and biomarkers of treatment response. To advance clinical translation, future studies should prioritize the optimization of dosing regimens, the evaluation of alternative nanocarriers, and the systematic comparison of administration routes. Comprehensive toxicity and posology studies are also essential. Furthermore, a deeper understanding of how polyphenols modulate key cellular processes—such as autophagy, inflammation, and apoptosis—will be crucial for maximizing their therapeutic potential while minimizing adverse interactions.

Finally, it is important to acknowledge the potential discrepancies between in vitro and in vivo results. A major limitation of the current literature is the lack of reproducibility, particularly in animal studies, due to inconsistencies in experimental design, compound concentrations, and methodology. These variations hinder cross-study comparisons and reduce the reliability of individual findings, but they also underscore the need for standardized and rigorously controlled protocols. In conclusion, while quercetin, luteolin, and apigenin exhibit promising antitumoral properties against glioma, further research is needed to overcome pharmacokinetic barriers, improve delivery systems, and ensure their safe and effective integration into multimodal treatment regimens. With continued scientific effort, these natural compounds may become valuable adjuncts in the therapeutic arsenal against gliomas.

## Figures and Tables

**Table 1:** Summarized results from in vivo studies investigating the use of luteolin in the treatment of gliomas.

Dosage	Animal Species	Tumor Type	Way of Administration	Time Period	Results	Ref.
50 mg/kg	Female nude BALB/c mice	Glioma (C6 cell line) Glioblastoma (U87 cell line)	Intravenous injection (1 time a day)	The administration of luteolin was continued for 15 days	C6 glioma group: ↓ tumor volume U87 glioma group: ↓ tumor volume	[30]
5 μg/mL	Zebrafish	Glioblastoma (U87 cell line)	Maintaining proper luteolin concentration in zebrafish incubating solution	Proper concentration of luteolin in zebrafish incubating solution was maintained for 5 days	↓ tumor volume	[30]
50 mg/kg	C57 mice	Glioma (GL261 cell line)	Intravenous injection (1 time a day)	The administration of luteolin was continued for 13 days	↓ tumor volume ↓ CD31 expression in tumor cells ↓ angiogenesis in tumor tissue ↑ apoptosis of tumor cells	[32]
10 mg/kg	Male BALB/c athymic nude mice	Glioblastoma (U87MG cell line)	Intraperitoneal injection (1 time every 2 days)	The administration of luteolin was started once tumors reached volumes of 70–100 cm^3^ and continued until day 35 of the whole experiment	↓ tumor growth through the activation of caspase-3 and cleaved capsase-12 in tumor cells ↑ endoplasmic reticulum stress through ATF4 and CHOP proteins in tumor cells	[67]

↓ means decreased; ↑ means increased.

**Table 2:** Summarized results from in vivo studies investigating the use of quercetin in the treatment of gliomas.

Dosage	Animal Species	Tumor Type	Way of Administration	Time Period	Results	Ref.
100 mg/kg	Male nude mice	Glioblastoma (U87 cell line)	Intraperitoneal injection (1 time a day)	The administration of quercetin was started once tumors reached volume of 100 cm^3^ and continued until the day 21 of whole experiment	↓ tumor volume ↓ Ki67-positive cells number in tumor tissue ↓ expression of *N*-cadherin, vimentin, p-GSK-3β, β-catenin, and ZEB1 in tumor cells ↑ expression of E-cadherin in tumor cells ↓ mice weight loss	[98]
1.5 mg/kg (encapsulated in liposomes with 3-BP	Male Sprague–Dawley rats	Glioma (C6 cell line)	Intraperitoneal injection (1 time every 3 days)	The administration of quercetin was continued for 6 days	↓ angiogenesis in tumor tissue ↓ tumor volume	[31]
50 mg/kg	Male Wistar rats	Glioma (C6 cell line)	Intraperitoneal injection (1 time a day)	The administration of quercetin was continued for 15 days	↓ lymphocytic infiltration in tumor tissue ↓ T-cell proliferation	[96]
25 mg/kg (encapsulated in FD-NMs or NLs)	BALB/c nude mice	Glioma (C6 cell line)	Intragastric administration (1 time every 7 days)	The administration of quercetin was continued for 28 days	FD-NMs group: ↓ tumor growth rate ↓ tumor volume ↑ survival time ↓ Bcl-2 expression in tumor cells NLs group: ↓ tumor growth rate ↓ tumor volume	[91]
25 mg/kg (in form of polyphenol nanoparticles)	Female C57BL/6 mice Athymic NCr-nu/nu mice	Glioma (GL261 cell line) Glioblastoma (PS30 cell line)	Intravenous injections (2 times per week)	The administration of quercetin was continued for 21 days	↓ tumor growth rate ↑ survival time ↑ vessel loss and cellular apoptosis in tumor tissues	[99]

↓ means decreased; ↑ means increased.

**Table 3:** Summarized results from in vivo studies investigating the use of apigenin in the treatment of gliomas.

Dosage	Animal Species	Tumor Type	Way of Administration	Time Period	Results	Ref.
50 mg/kg	Adult athymic mice	Glioma (C6 cell line)	Intratumoral injection (3 times per day)	The administration of apigenin was continued for 12 days	↓ tumor volume (modest)	[104]
20 mg/kg	Mice	Glioblastoma (SU3-5R cell line)	Intraperitoneal injection (1 time a day)	The administration of apigenin was continued for 12 days	↓ expression of NF-κB, HIF-1α, GLUT-1, GLUT-3, PKM2 ↓ activity of glycolytic enzymes ↑ susceptibility to radiation at dose of 8 gray	[122]

↓ means decreased; ↑ means increased.
